# Full population results from the core phase of CompLEEment-1, a phase 3b study of ribociclib plus letrozole as first-line therapy for advanced breast cancer in an expanded population

**DOI:** 10.1007/s10549-021-06334-0

**Published:** 2021-08-19

**Authors:** Michelino De Laurentiis, Simona Borstnar, Mario Campone, Ellen Warner, Javier Salvador Bofill, William Jacot, Susan Dent, Miguel Martin, Alistair Ring, Paul Cottu, Janice Lu, Eva Ciruelos, Hamdy A. Azim, Sanjoy Chatterjee, Katie Zhou, Jiwen Wu, Lakshmi Menon-Singh, Claudio Zamagni

**Affiliations:** 1 Division of Breast Medical Oncology, Department of Breast and Thoracic Oncology Director, Istituto Nazionale Tumori IRCCS “Fondazione Pascale”, Napoli, Italy; 2 Institute of Oncology Ljubljana, Ljubljana, Slovenia; 3 Western Cancer Institute, Nantes, France; 4 Sunnybrook Health Sciences Centre, Toronto, ON Canada; 5 Virgen del Rocío University Hospital, Biomedicine Institute, Seville, Spain; 6 Montpellier Cancer Institute, Montpellier, France; 7 The Ottawa Hospital Cancer Centre, Ottawa, ON Canada; 8 Gregorio Marañón General University Hospital, GEICAM, Universidad Complutense, CIBERONC, Madrid, Spain; 9 Royal Marsden Hospital NHS Foundation Trust, Sutton, UK; 10 Curie Institute, Paris, France; 11 USC Norris Comprehensive Cancer Center, Los Angeles, CA USA; 12 University Hospital 12 de Octubre, Clara Campal Comprehensive Cancer Center (HM CIOCC), Madrid, Spain; 13 Faculty of Medicine, Cairo University, Cairo, Egypt; 14 Tata Medical Center, Kolkata, India; 15 Novartis Pharmaceuticals Corporation, East Hanover, NJ USA; 16 Azienda Ospedaliero-Universitaria Di Bologna, Bologna, Italia; 17 Duke Cancer Institute, Duke University School of Medicine, Durham, NC USA

**Keywords:** Advanced breast cancer, CDK4/6 inhibitor, Endocrine therapy, Ribociclib

## Abstract

**Purpose:**

CompLEEment-1 is a phase 3b trial in an expanded patient population with hormone receptor-positive (HR +), human epidermal growth factor receptor-2–negative (HER2–) advanced breast cancer (ABC), the largest current trial of cyclin-dependent kinase 4 and 6 inhibitors in ABC.

**Methods:**

Patients treated with ≤ 1 line of prior chemotherapy and no prior endocrine therapy for ABC received ribociclib 600 mg/day (3-weeks-on/1-week-off) plus letrozole 2.5 mg/day and additionally monthly goserelin/leuprolide in men and pre-/perimenopausal women. Eligibility criteria allowed inclusion of patients with stable CNS metastases and an Eastern Cooperative Oncology Group performance status of 2. Primary objectives were safety and tolerability, and secondary objectives were efficacy and quality of life (QoL).

**Results:**

Overall, 3,246 patients were evaluated (median follow-up 25.4 months). Rates of all-grade and grade ≥ 3 treatment-related adverse events (AEs) were 95.2% and 67.5%, respectively. Treatment-related discontinuations due to all grade and grade ≥ 3 AEs occurred in 12.9% and 7.3% of patients, respectively. Rates of all-grade AEs of special interest (AESI) were as follows: neutropenia (74.5%), increased alanine aminotransferase (16.2%), increased aspartate aminotransferase (14.1%), and QTcF prolongation (6.7%); corresponding values for grade ≥ 3 AESI were 57.2%, 7.7%, 5.7%, and 1.0%, respectively. Median time to progression was 27.1 months (95% confidence interval, 25.7 to not reached). Patient QoL was maintained during treatment.

**Conclusion:**

Safety and efficacy data in this expanded population were consistent with the MONALEESA-2 and MONALEESA-7 trials and support the use of ribociclib plus letrozole in the first-line setting for patients with HR + , HER2– ABC.

**Trial registration:**

linicalTrials.gov NCT02941926.

**Supplementary Information:**

The online version contains supplementary material available at 10.1007/s10549-021-06334-0.

## Introduction

Approximately 75% of advanced breast cancers (ABCs) are hormone receptor‑positive (HR +) [1–3]. For many years, endocrine therapy (ET) has been the treatment of choice for patients with HR + ABC; however, resistance remains a barrier to long-term clinical benefit, leading to the development of therapies that reverse or delay this resistance [4]. The combination of ET with cyclin-dependent kinases 4/6 inhibitors (CDK4/6is) is the recommended first-line treatment for patients with HR + , human epidermal growth factor receptor-2–negative (HER2–) ABC, in the absence of visceral crisis [5, 6].

In multiple phase 3 clinical trials in HR + , HER2– ABC, ribociclib (a selective, small-molecule CDK4/6i) in combination with ET has demonstrated superior clinical benefit compared with endocrine monotherapy, including significantly better overall survival (OS) in premenopausal women when given with a non-steroidal aromatase inhibitor (NSAI) (MONALEESA-7) [7] and in postmenopausal women in combination with fulvestrant (MONALEESA-3) [8]. The ongoing phase 3 MONALEESA-2 study is investigating ribociclib with letrozole vs letrozole alone in postmenopausal women. Although OS results are awaited, MONALEESA-2 has reported a significant improvement in progression-free survival (PFS) in the ribociclib arm (25.3 months vs 16.0 months, hazard ratio = 0.568; 95% confidence interval [CI], 0.457–0.704; *P* = 9.63 × 10^–8^) [9]. Clinical trials, however, are often limited to patients with good performance status and frequently exclude patients with central nervous system (CNS) metastases and patients who have received chemotherapy for ABC [10, 11]. Furthermore, some patient populations (e.g., men with breast cancer) are small and their representation in ABC trials is uncommon [12].

Data from clinical practice indicate that a significant proportion of patients with HR + , HER2– ABC still receive chemotherapy as first-line treatment [13–16], while international guidelines recommend this only in the presence of visceral crisis [5]. Notably, premenopausal women and those with visceral or CNS metastases are understood to have worse outcomes when treated with ET and may be more likely to receive chemotherapy as first-line treatment for HR + , HER2– ABC [16]. Further information is needed to better understand the safety and efficacy of ET plus CDK4/6is in these patients.

The CompLEEment-1 trial investigated the safety and efficacy of ribociclib in combination with letrozole in a large, diverse patient cohort, representative of real‑world clinical practice, who had not received prior ET for advanced disease. The trial included a Core Phase (time from first patient/first visit to 18 months after the last patient/first visit) and a follow-on Extension Phase to last patient/last visit. Here, we report safety and efficacy results from the Core Phase.

### Methods

## Study design and treatment

CompLEEment-1 is a multicenter phase 3b trial (see Online Resource 1) designed to evaluate the overall safety, tolerability, and clinical efficacy of ribociclib in combination with letrozole in pre-/postmenopausal women and men with HR + , HER2− ABC and no prior ET for advanced disease. Patients were treated with ribociclib (starting dose: 600 mg orally, once daily, 3-weeks-on/1-week-off) plus letrozole (2.5 mg orally, once daily, on a continuous schedule) with or without food. Pre-/perimenopausal women and men also received goserelin (3.6 mg subcutaneously) or leuprolide (7.5 mg intramuscularly; added per protocol amendment in response to guideline updates [6, 17], administered on Day 1 of Cycle 1 and every 28 days thereafter). Treatment continued until disease progression, unacceptable toxicity, death, or discontinuation from study treatment for any other reason. Patients who discontinued ribociclib were discontinued from the study and censored. All patients were followed for 30 days following the last ribociclib dose. The Supplement includes further information on criteria for dose interruption, reduction, or permanent discontinuation due to adverse events (AEs) (see Online Resources 8–11), medications prohibited during this study (See Online Resource 12), and patient selection criteria.

### Endpoints

The primary endpoint was safety/tolerability, measured by the number of patients who experienced: any AEs; grade 3/4 AEs; serious AEs (SAEs); AEs of special interest (AESI); AEs leading to dose reduction, interruption, or discontinuation; and AE-related deaths. AESIs were defined according to ongoing reviews of all ribociclib safety data, including neutropenia, QT interval corrected for heart rate using Fridericia’s formula (QTcF) prolongation, and hepatobiliary toxicity. In an exploratory analysis, exposure-adjusted AEs were also evaluated. Acceptable safety/tolerability constituted similar results to those observed in the MONALEESA trials.

Secondary endpoints related to efficacy were time to progression (TTP) based on investigator assessment, overall response rate (ORR) for patients with measurable disease, and clinical benefit rate (CBR); PFS, although not included as a predefined endpoint, was evaluated. Efficacy and response classifications are defined in Online Resource 2. Other secondary endpoints included patient-reported outcome (PRO) measures of health-related quality of life (HRQoL) using the Functional Assessment of Cancer Therapy–Breast Cancer (FACT-B) questionnaire; due to the nature of the questionnaire and validation, it was only completed by female patients. Data were collected electronically in pre-selected countries: the USA, Canada, the UK, France, Italy, and Spain. FACT-B data were entered by patients at the clinic.

### Assessments

Safety was monitored by assessing patient symptoms through physical exams, including measurement of vital signs, cardiac assessment (i.e., 12-lead electrocardiogram), and assessing biochemical and hematologic laboratory values at various timepoints during the Core Phase; details of the assessment schedule are shown in Online Resource 2. AEs were characterized and graded according to National Cancer Institute Common Terminology Criteria for Adverse Events (CTCAE), v4.03 [18].

Tumor response was assessed locally, based on Response Evaluation Criteria in Solid Tumors (RECIST) v1.1. Tumor assessments were performed according to the current standard of care; assessments are recommended to take place every 12 weeks until disease progression (See Online Resource 2). There was no planned central review of imaging assessments.

### Statistical analysis

The safety analysis and full analysis sets were used for statistical analysis and data reporting. The safety analysis (safety outcomes) and full analysis (efficacy outcomes) sets comprised patients who received ≥ 1 dose of either ribociclib or letrozole or goserelin/leuprolide (if applicable) in the Core Phase.

The primary endpoint of safety/tolerability (number [%] of AEs, grade 3/4 AEs, and SAEs, AESI and AEs leading to treatment discontinuation and deaths, and AEs leading to dose reduction or dose interruption) was summarized descriptively in the safety analysis set. Attempts were made to ensure that comprehensive information was obtained for safety data and no imputation was applied for missing data. Data obtained for relative dose intensity (RDI) considered both zero and non-zero dose days and therefore accounted for dose interruptions.

For the secondary endpoint of TTP and for the post hoc, exploratory analysis of PFS, distribution was estimated using the Kaplan–Meier method and medians were presented with 95% CIs [19]. The other secondary endpoints of ORR and CBR were summarized using descriptive statistics (N [%]) combined with 2-sided exact binomial 95% CIs [20].

The PRO analysis set comprised female patients in pre-selected countries for whom baseline and ≥ 1 post-baseline PRO measurements were available. Descriptive statistics were used to summarize the FACT-B questionnaire subscale and overall scores at each scheduled assessment; the change from baseline at each assessment was summarized. Scores for a given scale/subscale were considered missing if > 50% of the items were missing; otherwise, the average of the non-missing items was used to impute missing items.

## Results

### Study population and disposition

Overall, 3,246 patients were enrolled and received ≥ 1 dose of study treatment between November 30, 2016 and March 22, 2018. The cut-off date for this analysis was November 8, 2019, and the median duration of follow-up was 25.4 months (minimum 19.1 months).

At data cutoff, 1,301 (40.1%) patients had completed Core Phase treatment, 415 of whom moved to the Extension Phase. Overall, 1,945 (59.9%) patients permanently discontinued treatment, mostly due to progressive disease (34.2%) and AEs (15.5%).

Patient age ranged from 20 to 92 years (median, 58.0 years). Patient populations of special interest, including patients ≥ 75 years of age, premenopausal female patients, male patients, patients with *de novo* ABC, patients with CNS metastases, patients with an Eastern Cooperative Oncology Group (ECOG) performance status 2, and patients who had received prior chemotherapy for ABC, are described in Table 1. At baseline, 2079 (64.0%) patients had measurable disease.

**Table 1:** Demographic and baseline characteristics of the CompLEEment-1 study population

Demographic variable	All patients (*N* = 3,246)
Age (years), median (range)	58.0 (20–92)
Age category (years), *n* (%)	
< 65	2,173 (66.9)
65 to < 70	440 (13.6)
70 to < 75	324 (10.0)
≥ 75	309 (9.5)
Gender, *n* (%)	
Male	39 (1.2)
Female, premenopausal	722 (22.2)
Female, postmenopausal	2,485 (76.6)
Race, *n* (%)	
Caucasian	2,553 (78.7)
Asian	227 (7.0)
Black	29 (0.9)
Native American	18 (0.6)
Pacific Islander	1 (0.03)
Other or unknown	418 (12.9)
Body mass index (kg/m^2^), mean (SD)	26.8 (5.6)
ECOG performance status, *n* (%)	
0	1,964 (60.5)
1	1,161 (35.8)
2	112 (3.5)
Missing	9 (0.3)
Hormone receptor status, *n* (%)	
Estrogen receptor-positive	3,231 (99.5)
Estrogen receptor-negative	15 (0.5)
Progesterone receptor-positive	2,608 (80.3)
Progesterone receptor-negative	574 (17.7)
Progesterone receptor unknown	64 (2.0)
HER2 receptor status, *n* (%)	
Negative	3,244 (99.9)
Positive^a^	2 (0.1)
Histological grade, *n* (%)	
Well differentiated	297 (9.1)
Moderately differentiated	1,306 (40.2)
Poorly differentiated	626 (19.3)
Undifferentiated	30 (0.9)
Unknown or missing	987 (30.4)
Prior (neo) adjuvant ET, *n* (%)	
Fulvestrant	4 (0.1)
Tamoxifen	1,153 (35.5)
Toremifene	3 (0.1)
Anastrozole	322 (9.9)
Exemestane	143 (4.4)
Letrozole	626 (19.3)
Disease-free interval, *n* (%)	
*De novo*^b^	1,041 (32.1)
Non-*de novo*^c^	2,201 (67.8)
≤ 24 months	382 (11.8)
> 24 months	1,819 (56.0)
Missing	4 (0.1)
Metastatic sites, *n* (%)	
0	15 (0.5)
1	903 (27.8)
2	923 (28.4)
3	644 (19.8)
4	375 (11.6)
≥ 5	386 (11.9)
Site of metastases, *n* (%)	
Bone	2,409 (74.2)
Bone only	704 (21.7)
Breast	183 (5.6)
CNS	51 (1.6)
Visceral	1,992 (61.4)
Liver	862 (26.6)
Lung	1,416 (43.6)
Other	295 (9.1)
Skin	110 (3.4)
Lymph nodes	1,250 (38.5)
Other	163 (5.0)
Chemotherapy for advanced disease, *n* (%)	324 (10.0)

*CNS* central nervous system, *ECOG* Eastern Cooperative Oncology Group, *ET* endocrine therapy, *HER2* human epidermal growth factor receptor-2, *SD* standard deviation

^a^After HER2 + status was confirmed, both patients were discontinued due to protocol deviation

^b^*De novo* includes patients with no date of first recurrence/progression or with a first recurrence/progression within 90 days of initial diagnosis without prior antineoplastic medication

^c^Non-*de novo* disease was calculated as the time from initial diagnosis to first recurrence/progression, categorized as ≤ 12 months, > 12 to ≤ 24 months, and ≥ 24 months

The median duration of exposure to ribociclib was 17.5 months and the median and mean RDIs were 95.2% and 86.4%, respectively. The median duration of exposure to letrozole was 17.7 months. The average daily dose of ribociclib was 547.7 mg (median 600.0 mg).

### Primary endpoint

At the data cut-off date, 3,203 (98.7%) patients had experienced an AE and 2,461 (75.8%) patients had experienced a grade ≥ 3 AE. The most common (≥ 20%) all‑grade AEs (regardless of causality) were neutropenia (*n* = 2,417; 74.5%), nausea (*n* = 1166; 35.9%), leukopenia (*n* = 887; 27.3%), fatigue (*n* = 760; 23.4%), diarrhea (*n* = 690; 21.3%), arthralgia (*n* = 677; 20.9%), and vomiting (*n* = 649; 20.0%), whereas the most common (≥ 5%) all-cause grade ≥ 3 AEs were neutropenia (*n* = 1856; 57.2%), leukopenia (*n* = 345; 10.6%), alanine aminotransferase (ALT) increased (*n* = 249; 7.7%), and aspartate aminotransferase (AST) increased (*n* = 184; 5.7%) (Table 2). Thirty-six (1.1%) patients experienced febrile neutropenia. The number of patients who discontinued due to an AE (regardless of causality) was 528 (16.3%), with the most common AEs (regardless of causality) causing discontinuation being ALT increased (197; 6.1%), AST increased (129; 4.0%), and transaminases increased (22; 0.7%).

**Table 2:** Overview of adverse events occurring in > 5% of patients (safety analysis set)

AEs, *n* (%)	All patients (*N* = 3,246)	
	All grades	Grade ≥ 3
AE overview		
AEs	3,203 (98.7)	2,461 (75.8)
Treatment related	3,091 (95.2)	2,192 (67.5)
SAEs	702 (21.6)	590 (18.2)
Treatment related	203 (6.3)	178 (5.5)
Fatal SAEs	62 (1.9)	61 (1.9)
Treatment related	14 (0.4)	14 (0.4)
AEs leading to discontinuation	528 (16.3)	310 (9.6)
Treatment related	418 (12.9)	237 (7.3)
AEs leading to dose adjustment/interruption	2,434 (75.0)	2,095 (64.5)
Treatment related	2,235 (68.9)	1,964 (60.5)
AEs requiring additional therapy	2,624 (80.8)	844 (26.0)
Treatment related	1,613 (49.7)	392 (12.1)
AEs by preferred term		
Neutropenia^a^	2,417 (74.5)	1,856 (57.2)
Nausea	1,166 (35.9)	26 (0.8)
Leukopenia^b^	887 (27.3)	345 (10.6)
Fatigue	760 (23.4)	49 (1.5)
Diarrhea	690 (21.3)	47 (1.4)
Arthralgia	677 (20.9)	14 (0.4)
Vomiting	649 (20.0)	34 (1.0)
Alopecia	638 (19.7)	0
Asthenia	632 (19.5)	34 (1.0)
Anemia	605 (18.6)	94 (2.9)
Constipation	554 (17.1)	11 (0.3)
ALT increased	526 (16.2)	249 (7.7)
Cough	493 (15.2)	4 (0.1)
Hot flush	490 (15.1)	5 (0.2)
Headache	462 (14.2)	15 (0.5)
AST increased	459 (14.1)	184 (5.7)
Back pain	437 (13.5)	29 (0.9)
Pruritus	431 (13.3)	10 (0.3)
Pyrexia	415 (12.8)	21 (0.6)
Decreased appetite	402 (12.4)	13 (0.4)
Rash	374 (11.5)	21 (0.6)
Dyspnea	308 (9.5)	48 (1.5)
Stomatitis	292 (9.0)	7 (0.2)
Edema peripheral	280 (8.6)	5 (0.2)
Insomnia	274 (8.4)	2 (0.1)
Pain in extremity	268 (8.6)	10 (0.3)
Thrombocytopenia	256 (7.9)	38 (1.2)
Dizziness	239 (7.4)	5 (0.2)
Dyspepsia	237 (7.3)	0
Bone pain	228 (7.0)	12 (0.4)
Dry skin	228 (7.0)	3 (0.1)
Hypertension	227 (7.0)	68 (2.1)
Abdominal pain	221 (6.8)	19 (0.6)
Electrocardiogram QTcF prolonged	217 (6.7)	33 (1.0)
Abdominal pain upper	216 (6.7)	7 (0.2)
Urinary tract infection	208 (6.4)	20 (0.6)
Musculoskeletal pain	205 (6.3)	7 (0.2)
Blood creatinine increased	201 (6.2)	4 (0.1)
Upper respiratory tract infection	196 (6.0)	7 (0.2)
Nasopharyngitis	194 (6.0)	0
Myalgia	192 (5.9)	0
Lymphopenia	166 (5.1)	88 (2.7)

A patient with multiple severity grades for an AE was only counted under the maximum grade

*AE* adverse event, *ALT* alanine aminotransferase, *AST* aspartate aminotransferase, *QTcF* QT interval corrected for heart rate using Fridericia’s formula, *SAE* serious adverse event

^a^Includes “neutropenia” and “neutrophil count decreased”

^b^Includes “leukopenia” and “white blood cell count decreased”

In total, 3091 (95.2%) patients experienced a treatment-related AE, 2192 (67.5%) patients experienced a grade ≥ 3 treatment-related AE, and 203 (6.3%) patients experienced a treatment-related SAE (Table 2). Treatment-related all grade and grade ≥ 3 AEs led to dose adjustment/interruption for 2,235 (68.9%) and 1,964 (60.5%) patients, respectively. In total, 418 (12.9%) patients had treatment-related AEs (regardless of causality) leading to discontinuation, and 237 (7.3%) patients had grade ≥ 3 treatment-related AEs leading to discontinuation. Treatment-related grade 5 SAEs are presented in Online Resource 13.

Values for all grade and grade ≥ 3 AESIs, respectively, were as follows: 217 patients (6.7%) and 33 patients (1.0%) experienced QTcF prolongation, 526 patients (16.2%) and 249 patients (7.7%) experienced increased ALT, and 459 patients (14.1%) and 184 patients (5.7%) experienced increased AST (neutropenia values have already been stated) (See Online Resource 14). A > 60 ms increase from baseline in QTcF interval was observed in 189 (5.9%) patients, whereas a new QTcF of > 480 to ≤ 500 ms and > 500 ms was observed in 59 (1.8%) and 42 (1.3%) patients, respectively.

There were 1,716 (52.9%) patients and 597 (18.4%) patients who, because of neutropenia, required a ribociclib dose interruption or reduction, respectively. Otherwise, dose interruptions or reductions due to AESIs were rare. The numbers of patients who experienced permanent drug discontinuation because of neutropenia, ALT increased, AST increased, and QTcF prolongation were 18 (0.6%), 197 (6.1%), 129 (4.0%), and 8 (0.2%), respectively. At the data cutoff, the rate of recovery/resolution was greater than the rate of non-recovery/non-resolution for all AESIs (neutropenia, 70.1% vs 39.9%; ALT increased, 10.6% vs 8.0%; AST increased, 9.4% vs 7.1%; QTcF prolongation, 5.9% vs 1.0%). AESIs rarely led to hospitalization (0%–0.3%); no AESIs were fatal (Table 3). AESI events such as neutropenia, increased ALT, and increased AST, when adjusted for ribociclib exposure for time periods of 0–1 years, 1–2 years, and > 2 years, decreased in occurrence (Table 3). The median time to first occurrence of grade ≥ 3 neutropenia was 17.1 weeks (95% CI, 14.0–24.1). However, time to onset of grade ≥ 3 neutropenia decreased sharply after Week 8, and 60% event probability was not reached until after Week 80, while 70% event probability was not reached until after Week 128 (see Online Resource 3).

**Table 3:** Clinical impact of adverse events of special interest (safety analysis set)

AESI, *n* (%)^a^	Neutropenia^b^	ALT increased	AST increased	QTcF prolongation
All grade	2,417 (74.5)	526 (16.2)	459 (14.1)	217 (6.7)
Leading to dose interruption	1,716 (52.9)	245 (7.5)	209 (6.4)	39 (1.2)
Leading to dose reduction	597 (18.4)	50 (1.5)	29 (0.9)	20 (0.6)
Leading to drug withdrawal	18 (0.6)	197 (6.1)	129 (4.0)	8 (0.2)
Leading to hospitalization	6 (0.2)	9 (0.3)	7 (0.2)	0
Medication or therapy taken	114 (3.5)	62 (1.9)	54 (1.7)	7 (0.2)
Not recovered/not resolved	1,294 (39.9)	260 (8.0)	230 (7.1)	33 (1.0)
Recovering/resolving	1,160 (35.7)	213 (6.6)	147 (4.5)	21 (0.6)
Recovered/resolved	2,275 (70.1)	344 (10.6)	304 (9.4)	191 (5.9)
With sequelae	45 (1.4)	6 (0.2)	5 (0.2)	2 (0.1)
Leading to death	0	0	0	0
Exposure-adjusted occurrence rate, events per 100 patient years^c^				
All events	322.53	27.06	21.44	6.62
0–1 years	410.22	41.66	33.27	10.22
1–2 years	200.83	6.32	4.61	1.26
2 years	141.87	1.09	0.55	2.18

*AE* adverse event, *AESI* adverse event of special interest, *ALT* alanine aminotransferase, *AST* aspartate aminotransferase, *QTcF* QT interval corrected for heart rate using Fridericia’s formula

^a^Percentage value calculated based on 3,246 patients. A patient is counted no more than once in each AE outcome. If a patient has AEs with different outcomes, the patient will be counted in several outcomes. If the patient has several events with the same outcome, they will be counted only once in the corresponding outcome line

^b^Includes “neutropenia” and “neutrophil count decreased”

^c^Number of events divided by the corresponding sum of the exposure duration, where duration of exposure in patient-treatment years is duration of exposure in time interval

A total of 74 (2.3%) on-treatment deaths occurred up to the cut-off date (i.e., from the date of first administration of study treatment to 30 days after last administration of study treatment). ABC was the primary cause of death for 38 (1.2%) patients. Overall, 14 (0.4%) patients had a treatment-related fatal SAE; 22 deaths were unrelated to treatment.

### Secondary and exploratory endpoints

**Fig. 1:**
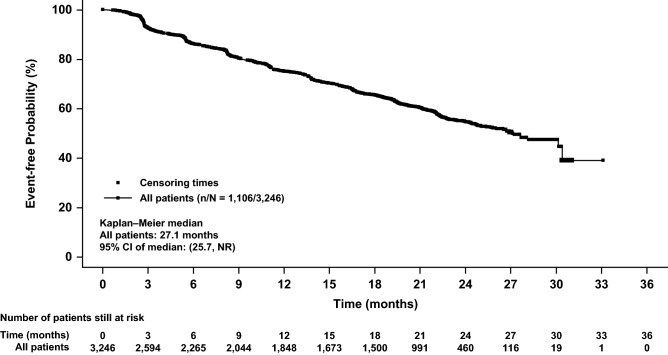
Kaplan–Meier plot of time to progression per local investigator assessment (full analysis set). *CI* confidence interval, *NR* not reached

Over a median follow-up of 25.4 months, the median TTP was 27.1 months (95% CI, 25.7 to not reached), whereas the estimated event-free probability at 24 months was 54.7% (95% CI, 52.5–56.8; Fig. 1). In total 2140 (65.9%) of patients were censored from the TTP analysis; the main reasons for censoring were ‘adequate assessment no longer available’ in 720 patients and ‘withdrawal of consent’ in 73 patients.

ORRs for the total population and patients with measurable disease at baseline (*n* = 2079 [64.0%]) were 29.3% (95% CI, 27.7–30.9) and 43.6% (95% CI, 41.5–45.8), respectively. The CBRs were similar for the total study population and patients with measurable disease at baseline (70.7% [95% CI, 69.1–72.2] and 69.1% [95% CI, 67.1–71.1], respectively) (Table 4). In an exploratory analysis, median PFS was 26.7 months (95% CI, 24.8–30.1) for the total study population (See Online Resource 4).

**Table 4:** Efficacy results as per local investigator assessment (full analysis set)

All patients, *n* (%)	*N* = 3,246
Best overall response	
CR	99 (3.0)
PR	851 (26.2)
Non-CR/Non-PD^a^	952 (29.3)
SD	813 (25.0)
PD	178 (5.5)
Unknown	353 (10.9)
ORR: CR + PR [95% CI]	950 (29.3) [27.7–30.9]
CBR: CR + PR + (SD + non-CR/non-PD ≥ 24 weeks) [95% CI]	2,294 (70.7) [69.1–72.2]
All patients, months (95% CI)	
Median PFS	26.7 (24.8–30.1)
Patients with measurable disease at baseline, *n* (%)	2,079 (64.0)
Best overall response^b^	
CR	56 (2.7)
PR	851 (40.9)
SD	810 (39.0)
PD	134 (6.4)
Unknown	228 (11.0)
ORR: CR + PR [95% CI]^b^	907 (43.6) [41.5–45.8]
CBR: CR + PR + (SD + non-CR/non-PD ≥ 24 weeks) [95% CI]^a,b^	1,437 (69.1) [67.1–71.1]

Response classifications are defined in Online Resource 2

*CBR* clinical benefit rate, *CI* confidence interval, *CR* complete response, *ORR* overall response rate, *PD* progressive disease, *PFS* progression‑free survival, *PR* partial response, *SD* stable disease

^a^Used instead of “Unknown” wherever possible (i.e., in situations where, based on available information, expert judgment could be used to identify equivocal progression [i.e., PD] or definitively rule this out)

^b^Percentages are based on population of patients with measurable disease at baseline

The PRO analysis set included 1,230 patients from 6 pre-selected countries. Descriptive analysis of median delay to first occurrence of a clinically relevant deterioration from baseline (≥ 7-point decrease) [21] in overall FACT-B score was not reached (See Online Resource 5). Overall changes in FACT-B scores from baseline until end of treatment were assessed, as well as FACT-B scores for individual domains, including physical, social/family, emotional and functional well-being, and additional concerns (See Online Resource 15). Scores for emotional and functional well-being did not decrease below baseline levels while on treatment (See Online Resources 6 and 7).

## Discussion

In the CompLEEment-1 study, ribociclib in combination with letrozole demonstrated safety and efficacy consistent with that seen in the pivotal phase 3 studies (MONALEESA-2 [9, 22] and MONALEESA-7 [7, 23]), in a much larger, diverse cohort of patients with HR + , HER2– ABC who had not previously received ET for advanced disease. The AE profile was manageable and no new safety signals were identified; in addition, median TTP was 27.1 months after a median follow-up of 25.4 months and patient HRQoL was not adversely impacted. The median PFS of 26.7 months is similar to that reported in the ribociclib arms of MONALEESA-2 (25.3 months [95% CI, 23.0–30.3]) [9] and MONALEESA-7 (23.8 months [95% CI, 19.2‑NR] [23]), indicating that the efficacy reported in phase 3 trials is achievable in real‑world practice.

The patient characteristics in CompLEEment-1 were more broadly representative of patients in clinical practice than those included in phase 3 trials of CDK4/6i combined with NSAI [7, 9, 24, 25]. The expanded patient population in CompLEEment-1 included patients treated with prior chemotherapy for advanced disease, patients with ECOG performance status 2, patients with stable CNS metastases, premenopausal women, and men—a larger population more representative of real‑world clinical practice, and one not well studied in randomized controlled trials with palbociclib and abemaciclib [22]. For example, 10.0% (324 patients) and 3.5% (112 patients) of those enrolled had received prior chemotherapy for advanced disease or had ECOG performance status 2, respectively. This represents a large group of patients and few trials have reported results in similarly broad populations. The study cohort was also diverse in terms of race and age. Compared with ribociclib-treated patients in MONALEESA-2, CompLEEment-1 included more patients with both disease-free interval ≤ 12 months (7.3% vs 1.2%) and ≥ 3 metastatic sites (43.3% vs 34.1%).

Many of the expected adverse reactions observed with ribociclib, including neutropenia, QTcF prolongation, and hepatobiliary adverse reactions, can be managed by following dose interruption or reduction guidelines as per the label and/or with medication [17, 26]. Although incidences of dose interruption and reduction due to neutropenia occurred in 52.9% and 18.4% of patients, respectively, incidences leading to drug withdrawal (0.6%) and additional medication (3.5%) were rare. Discontinuations because of AESIs were infrequent, and AESIs were resolved in most cases; hospitalizations resulting from these events were rare (< 1%). Furthermore, exposure-adjusted AE data suggest that occurrences of most AESIs decreased during ribociclib treatment.

Efficacy results relating to progression (TTP and PFS) in this study are conservative compared with those observed in MONALEESA-2, which included PFS events occurring after patients had discontinued ribociclib treatment (during post-treatment efficacy follow-up); these patients were censored in CompLEEment-1. Importantly, TTP only documents progression or death due to underlying cancer, whereas PFS documents progression or death due to any cause which precludes direct comparisons between these endpoints. The ORRs of the total population (29.3%) and patients with measurable disease (43.6%) are lower than those seen in ribociclib‑treated patients in MONALEESA-2 (42.5% in the total population, 54.5% in patients with measurable disease) and MONALEESA-7 (41% in the total population, 51% in patients with measurable disease) [7, 9, 23]. This may be due to certain between‑trial differences in baseline characteristics (e.g., prior chemotherapy exposure or more metastatic sites) that were less favorable in CompLEEment-1 vs the pivotal studies. However, when compared with the MONALEESA trials, lower ORRs did not appear to translate to shorter PFS in CompLEEment-1. A limitation of CompLEEment-1 is that response assessment timings allowed for different intervals according to the local standard of care. As with all prospective real-world studies, lack of randomization and a control arm necessitate the cautious interpretation of efficacy results.

Treatment guidelines recommend that, in addition to efficacy and safety, HRQoL is evaluated using validated PRO measures [5], e.g., the FACT-B questionnaire [27], which is frequently used in ABC trials. Baseline FACT-B domain scores were similar to those of other CDK4/6i trials in ABC [28]. The median delay to first occurrence of a clinically relevant deterioration (≥ 7-point decrease) in overall FACT-B score was not reached, implying that quality of life (QoL) was maintained while on treatment. Individual FACT-B domain scores for emotional and functional well-being did not appear to be impacted by treatment initiation and were maintained while on treatment, relative to baseline. Although QoL assessments must be interpreted cautiously in single-arm studies, overall FACT-B results suggest that patients’ HRQoL was maintained while taking ribociclib. This is consistent with QoL outcomes in MONALEESA-2 and complements the results observed in MONALEESA-7, the only CDK4/6i study with improved QoL in premenopausal patients [29, 30].

## Conclusion

Results from CompLEEment-1, the largest prospective dataset on ribociclib and any other CDK4/6i (to our knowledge), and one of the largest trials ever published in ABC, support the manageable safety profile and efficacy of ribociclib in combination with letrozole as first-line treatment for HR + , HER2– ABC. In addition to providing insights into the clinical management and mitigation of common AEs with ribociclib, CompLEEment-1 reports a vital assessment of a diverse patient population in conditions simulating a real-world setting. These results build on the available knowledge from the extensive MONALEESA program, and would potentially support clinical decisions in daily practice. Outcomes in patient subgroups of special interest will be presented in due course.

## Supplementary Information

Below is the link to the electronic supplementary material.

Supplementary file1 (pdf 428 KB)

## Data Availability

The data that support the findings of this study are available from Novartis Pharmaceuticals Corporation but restrictions apply to the availability of these data (https://www.novartisclinicaltrials.com/TrialConnectWeb/home.nov). Data are however available from the authors upon reasonable request and with permission of Novartis Pharmaceuticals Corporation.
