# Correction to: Full population results from the core phase of CompLEEment-1, a phase 3b study of ribociclib plus letrozole as first-line therapy for advanced breast cancer in an expanded population

**DOI:** 10.1007/s10549-021-06374-6

**Published:** 2021-10-08

**Authors:** Michelino De Laurentiis, Simona Borstnar, Mario Campone, Ellen Warner, Javier Salvador Bofill, William Jacot, Susan Dent, Miguel Martin, Alistair Ring, Paul Cottu, Janice Lu, Eva Ciruelos, Hamdy A. Azim, Sanjoy Chatterjee, Katie Zhou, Jiwen Wu, Lakshmi Menon-Singh, Claudio Zamagni

**Affiliations:** 1 Division of Breast Medical Oncology, Department of Breast and Thoracic Oncology Director, Istituto Nazionale Tumori IRCCS “Fondazione Pascale”, Napoli, Italy; 2 Institute of Oncology Ljubljana, Ljubljana, Slovenia; 3 Western Cancer Institute, Nantes, France; 4 Sunnybrook Health Sciences Centre, Toronto, ON Canada; 5 Virgen del Rocío University Hospital, Biomedicine Institute, Seville, Spain; 6 Montpellier Cancer Institute, Montpellier, France; 7 The Ottawa Hospital Cancer Centre, Ottawa, ON Canada; 8 Gregorio Marañón General University Hospital, GEICAM, Universidad Complutense, CIBERONC, Madrid, Spain; 9 Royal Marsden Hospital NHS Foundation Trust, Sutton, UK; 10 Curie Institute, Paris, France; 11 USC Norris Comprehensive Cancer Center, Los Angeles, CA USA; 12 University Hospital 12 de Octubre, Clara Campal Comprehensive Cancer Center (HM CIOCC), Madrid, Spain; 13 Faculty of Medicine, Cairo University, Cairo, Egypt; 14 Tata Medical Center, Kolkata, India; 15 Novartis Pharmaceuticals Corporation, East Hanover, NJ USA; 16 Azienda Ospedaliero-Universitaria Di Bologna, Bologna, Italia; 17 Duke Cancer Institute, Duke University School of Medicine, Durham, NC USA

## Correction to: Breast Cancer Research and Treatment 10.1007/s10549-021-06334-0

The article “Full population results from the core phase of CompLEEment‑1, a phase 3b study of ribociclib plus letrozole as first‑line therapy for advanced breast cancer in an expanded population”, written by Michelino De Laurentiis, Simona Borstnar, Mario Campone, Ellen Warner, Javier Salvador Bofill, William Jacot, Susan Dent, Miguel Martin, Alistair Ring, Paul Cottu, Janice Lu, Eva Ciruelos, Hamdy A. Azim, Sanjoy Chatterjee, Katie Zhou, Jiwen Wu, Lakshmi Menon‑Singh and Claudio Zamagni, was originally published electronically on the publisher’s internet portal on 19 August 2021 without open access. With the author(s)’ decision to opt for Open Choice the copyright of the article changed on 27 August 2021 to © The Author(s) 2021 and the article is forthwith distributed under a Creative Commons Attribution 4.0 International License, which permits use, sharing, adaptation, distribution and reproduction in any medium or format, as long as you give appropriate credit to the original author(s) and the source, provide a link to the Creative Commons licence, and indicate if changes were made. The images or other third party material in this article are included in the article’s Creative Commons licence, unless indicated otherwise in a credit line to the material. If material is not included in the article’s Creative Commons licence and your intended use is not permitted by statutory regulation or exceeds the permitted use, you will need to obtain permission directly from the copyright holder. To view a copy of this licence, visit http://creativecommons.org/licenses/by/4.0.

