# Supplementary material for: Full population results from the core phase of CompLEEment-1, a phase 3b study of ribociclib plus letrozole as first-line therapy for advanced breast cancer in an expanded population
Source: Breast Cancer Res Treat. 2021 Aug 19;189(3):689–99. doi: 10.1007/s10549-021-06334-0 (PMC8505291; doi:10.1007/s10549-021-06334-0)
Supplement: Supplementary file 1 — Supplementary file1 (pdf 428 KB) [file 10549_2021_6334_MOESM1_ESM.pdf]

Full population results from the core phase of CompLEEment-1, a phase 3b study of ribociclib plus letrozole as first-line therapy for advanced breast cancer in an expanded population

Michelino De Laurentiis, Simona Borstnar, Mario Campone, Ellen Warner, Javier Salvador Bofill, William Jacot, Susan Dent, Miguel Martin, Alistair Ring, Paul Cottu, Janice Lu, Eva Ciruelos, Hamdy A. Azim, Sanjoy Chatterjee, Katie Zhou, Jiwen Wu, Lakshmi Menon, Claudio Zamagni

Journal: ***Breast Cancer Research and Treatment***
Corresponding author email address: **m.delaurentiis@breastunit.org**

SUPPLEMENTARY MATERIAL

Study design and treatment

Dose interruptions and/or reductions for ribociclib (to 400 mg per day, with further reduction to 200 mg per day if needed) were permitted to manage treatment-related adverse events (see Online Resources 8–11); re-escalation of the dose of ribociclib was not permitted, and no dose reductions were allowed for letrozole, goserelin, or leuprolide. Prohibited medications during study drug treatment are shown in Online Resource 12 and included strong cytochrome P450 (CYP)3A4/5 inhibitors/inducers, CYP3A4/5 substrates with a narrow therapeutic index, and medications with a known risk for QT prolongation.

Patients

Adult women of any menopausal status and men with confirmed hormone receptor-positive, human epidermal growth factor 2-negative advanced breast cancer (ABC) (locoregionally recurrent or metastatic) not amenable to curative therapy were eligible. Patients with an Eastern Cooperative Oncology Group (ECOG) performance status of ≤2, a baseline QT interval corrected for heart rate using Fridericia's formula (QTcF interval) of <450 ms, and a resting heart rate of ≥50 beats per minute were eligible. Adequate bone marrow and organ function were required (as assessed by local laboratory), including absolute neutrophil count ≥1.5 × 10^9/L, alanine aminotransferase (ALT) and aspartate aminotransferase (AST) <2.5 × upper limit of normal (ULN) in the absence of liver metastases and <5 × ULN in patients with liver metastases.

Patients were ineligible if they had received prior endocrine therapy (ET) for ABC, a cyclin-dependent kinases 4 and 6 inhibitor, or >1 regimen of chemotherapy for ABC. Previous (neo)adjuvant therapy with a nonsteroidal aromatase inhibitor was not permitted unless the disease-free interval was >12 months from the end of standard adjuvant ET. Patients with central nervous system (CNS) metastases were not permitted unless (1) there had been ≥4 weeks from completion of therapy for CNS disease to the start of study treatment and (2) patients had clinically stable CNS lesions at the time of study treatment initiation and had not received steroids and/or enzyme-inducing anti-epileptic medications for the management of brain metastases for ≥2 weeks before study entry. Patients were ineligible if they had impaired gastrointestinal function or gastrointestinal disease that might significantly alter absorption of study drugs; had clinically significant, uncontrolled heart disease and/or cardiac repolarization abnormalities; or were taking medication(s) with a known risk for QT prolongation (eg, via drug–drug interaction, including those in Online Resource 12). A sample size of 3,000 patients was planned to detect rare adverse events with greater frequency and precision, and to allow meaningful safety analyses in subgroups of interest (male, ECOG PS=2, those with 1 prior line of chemotherapy).

Online Resource 1. CompLEEment-1 Study Design

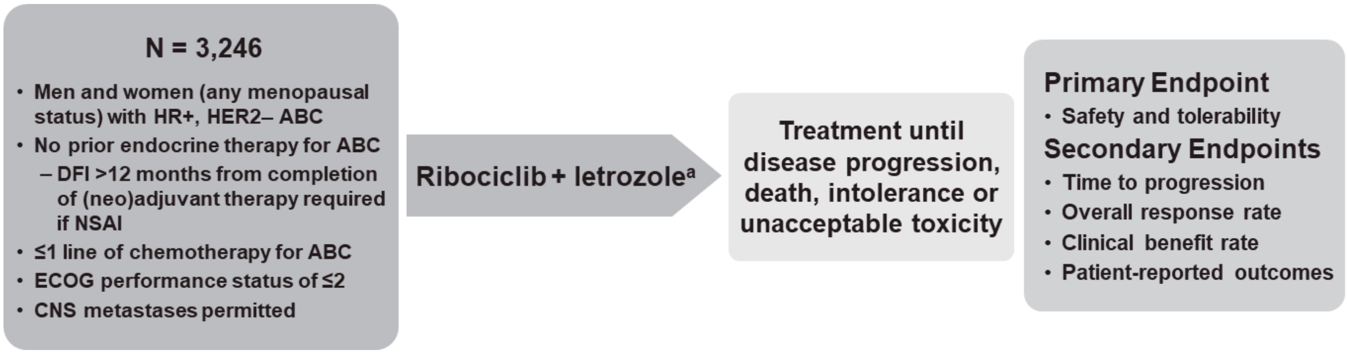

ABC, advanced breast cancer; CNS, central nervous system; DFI, disease-free interval; ECOG, Eastern Cooperative Oncology Group; HER2–, human epidermal growth factor receptor 2-negative; HR+, hormone receptor-positive; NSAI, nonsteroidal aromatase inhibitor. [a] Men and premenopausal women received goserelin during treatment; based on NCCN Breast Cancer Guidelines 2017 (National Comprehensive Cancer Network. NCCN Clinical Practice Guidelines in Oncology: Breast Cancer, version 2.2017), an amendment to the CompLEEment-1 protocol permitted the use of another luteinizing hormone-releasing hormone agonist (leuprolide, also administered monthly), in addition to goserelin.

Online Resource 2. CompLEEment-1 assessment schedule

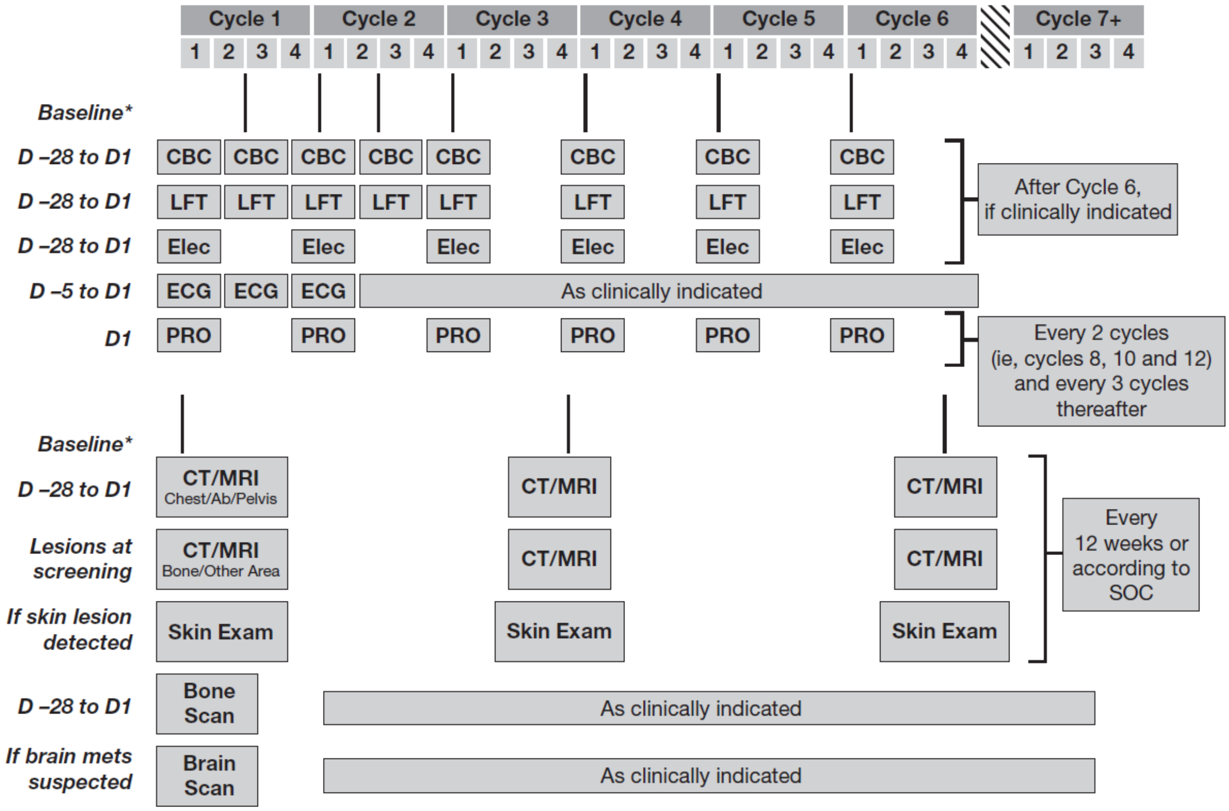

Overall response rate was defined as the proportion of patients with best overall response of complete response or partial response according to RECIST 1.1. Clinical benefit rate is defined as the proportion of patients with a best overall response of complete response or partial response, or an overall lesion response of stable disease, lasting as per local review for a duration of at least 24 weeks. Complete response was defined as disappearance of all non-nodal target lesions as well as a reduction in short axis to <10 mm for any pathological lymph nodes assigned as target lesions. Progressive disease was defined as a ≥20% increase in the sum of diameter of all measured target lesions, taking as reference the smallest sum of diameter of all target lesions recorded at or after baseline (in addition to the relative increase of 20%, the sum must also demonstrate an absolute increase of ≥5 mm). Partial response was defined as a ≥30% decrease in the sum of diameter of all target lesions, taking as reference the baseline sum of diameters. Stable disease was defined as insufficient shrinkage to qualify for partial response or complete response and no increase in lesions that would qualify for progressive disease.

Ab, abdomen; CBC, complete blood count; CT, computed tomography; D, day; ECG, electrocardiogram; Elec, electrolytes; LFT, liver function test; mets, metastases; MRI, magnetic resonance imaging; PRO, patient-reported outcomes; SOC, standard of care.

[a]The following assessments were required at screening/baseline: chest, abdomen, and pelvis CT or MRI; brain CT or MRI, if clinically indicated; whole-body bone scan; localized bone CT, MRI or X-ray, for any lesions identified on the whole-body bone scan that are not visible on the chest, abdomen, and pelvis CT or MRI; skin visual inspection and measurement (only if skin lesions at screening); CT or MRI of other metastatic sites (eg, neck), if clinically indicated.

Online Resource 3. Kaplan–Meier plot of time to onset of grade 3 or worse neutropenia (safety set)

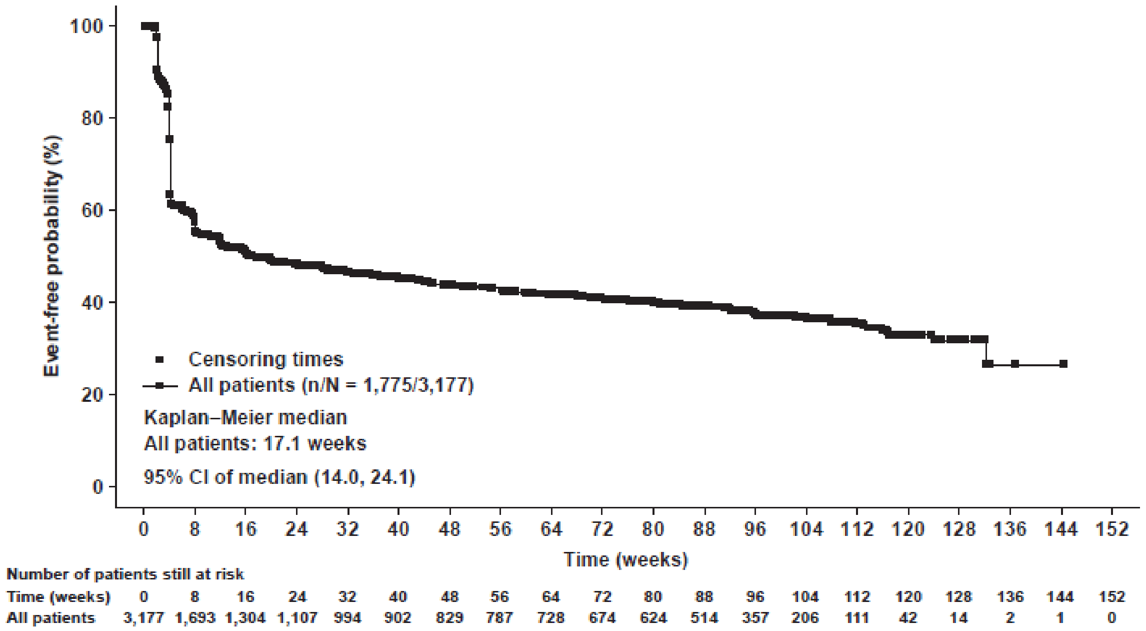

CI, confidence interval.

Online Resource 4. Kaplan–Meier plot of progression-free survival as per local investigator's assessment (full analysis set)

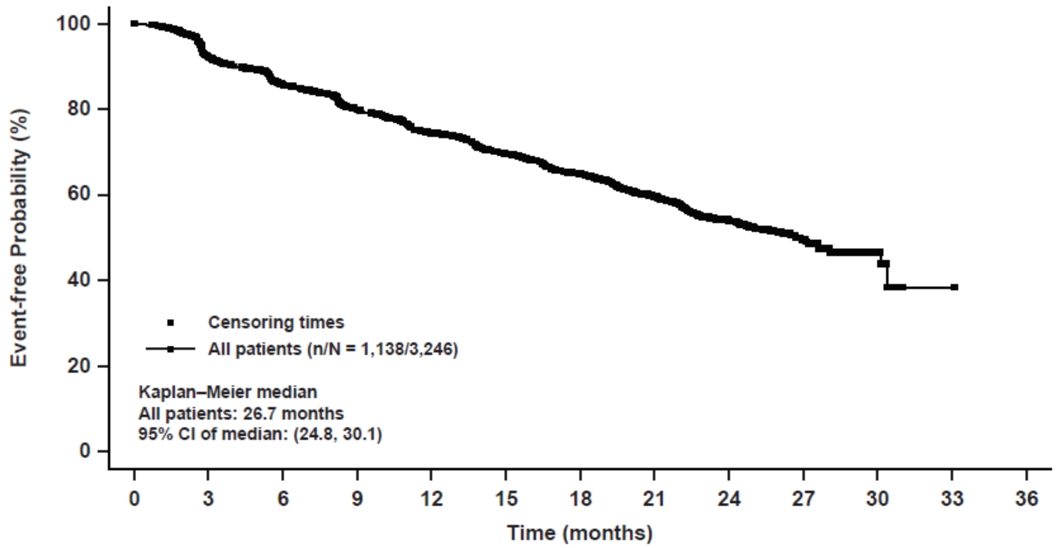

Number of patients still at risk

Time (months)	0	3	6	9	12	15	18	21	24	27	30	33	36
All patients	3,246	2,596	2,269	2,048	1,848	1,673	1,501	992	460	116	19	1	0

CI, confidence interval.

Online Resource 5. Kaplan–Meier plot of time to first occurrence of a clinically relevant deterioration, defined as a ≥7-point decrease in fact-b total scores (patient-reported outcomes analysis set)

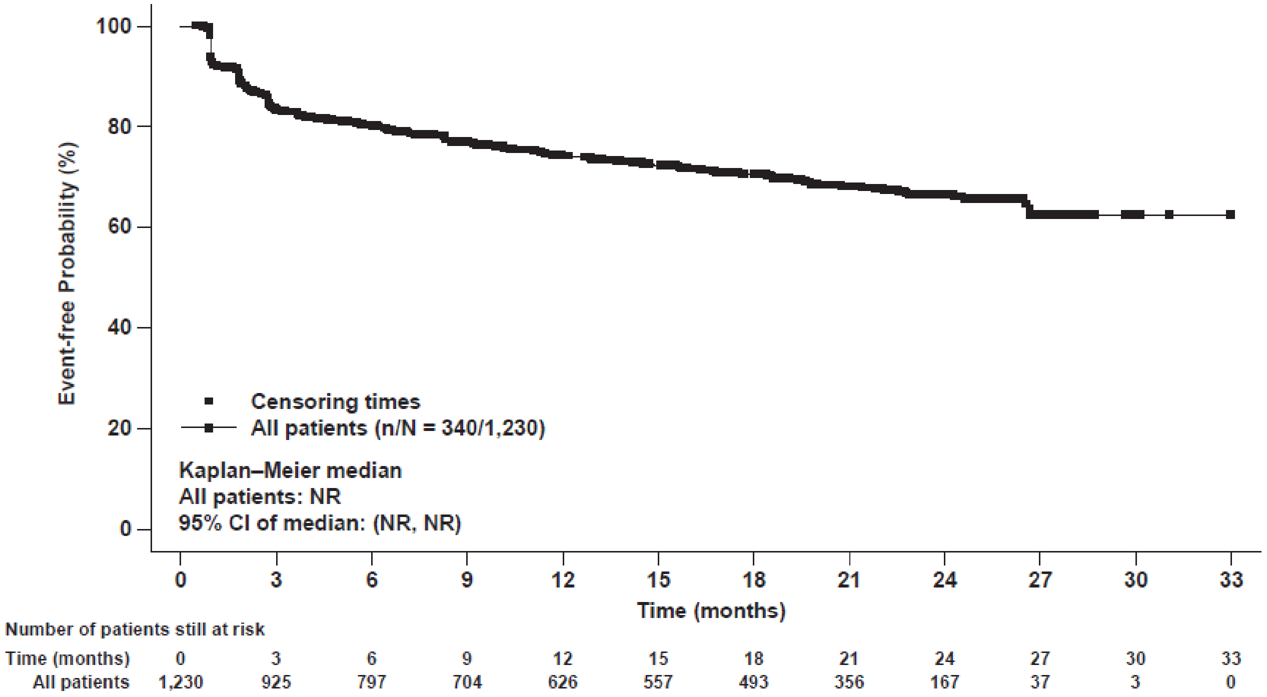

CI, confidence interval; NR, not reached.

Online Resource 6. Change from baseline in FACT-B emotional wellbeing scores (patient-reported outcomes analysis set)

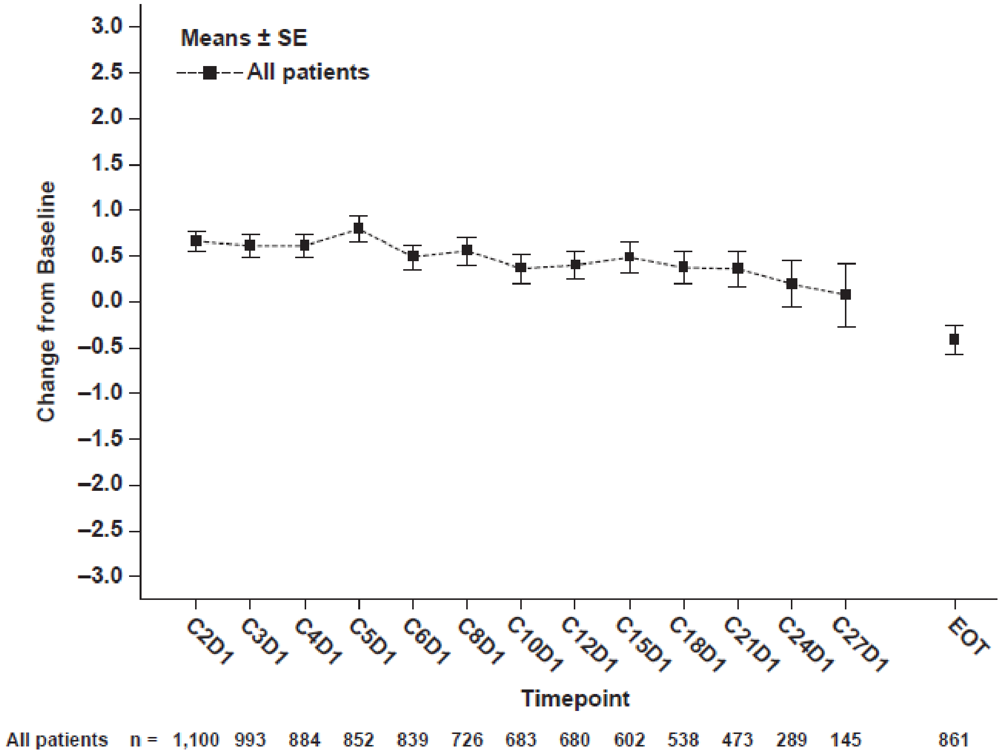

C, cycle; D, day; EOT, end of treatment; FACT-B, Functional Assessment of Cancer Therapy–Breast Cancer; SE, standard error of the mean.

Online Resource 7. Change from baseline in FACT-B functional wellbeing scores (patient-reported outcomes analysis set)

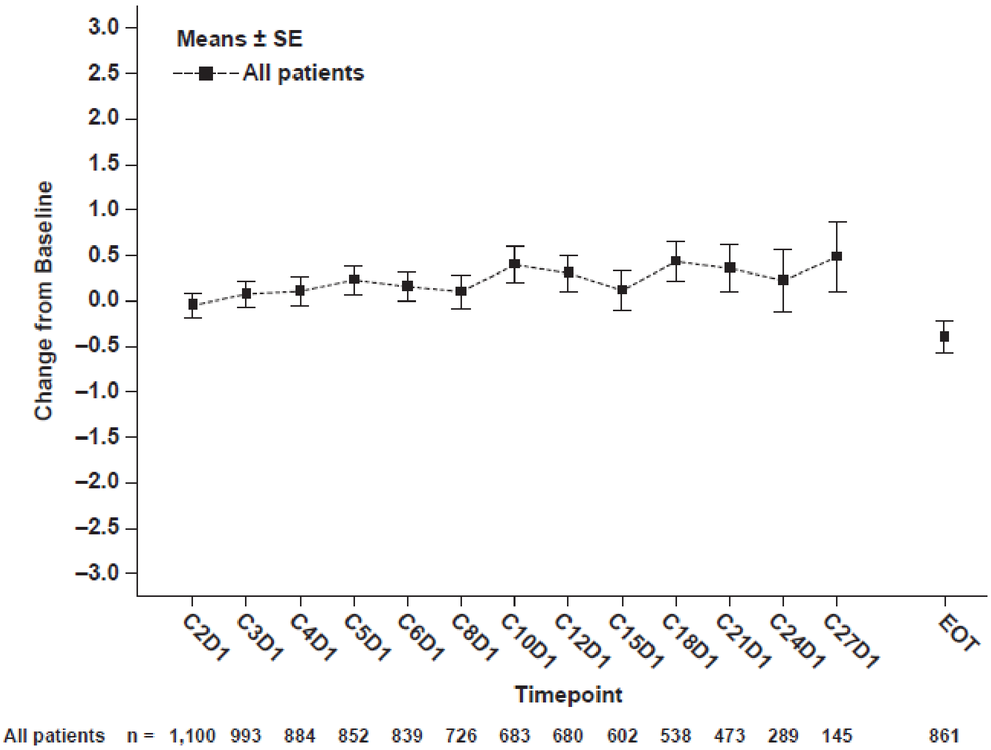

C, cycle; D, day; EOT, end of treatment; FACT-B, Functional Assessment of Cancer Therapy–Breast Cancer; SE, standard error of the mean.

Online Resource 8. Ribociclib dose reduction/interruption and management recommendations for most adverse reactions

Grade	Dose Adjustment and Management Recommendations
1	• No dose adjustment recommended. Initiate appropriate medical therapy and monitor
2	• Dose interruption until recovery to grade ≤1. Initiate appropriate medical therapy and monitor<br>• Re-initiate ribociclib at the same dose<br>• If the same toxicity recurs at grade 2, interrupt ribociclib until recovery to grade ≤1. Re-initiate ribociclib at the next lower dose level
3	• Dose interruption until recovery to grade ≤1. Initiate appropriate medical therapy and monitor<br>• Re-initiate ribociclib at the next lower dose level<br>• If the same toxicity recurs at grade 2: temporary dose interruption until recovery to grade ≤1 and reduce ribociclib dose the next lower dose level<br>• If toxicity recurs at grade 3, discontinue ribociclib
4	• Discontinue ribociclib and treat with appropriate medical therapy
Separate dose adjustment guidelines were available for hematological adverse drug reactions (See Online Resource 9), hepatic toxicities (See Online Resource 10), and QTcF prolongation (See Online Resource 11).	

QTcF, QT interval corrected for heart rate using Fridericia’s formula.

Online Resource 9. Ribociclib dose reduction/interruption and management recommendations for hematological adverse drug reactions

Toxicity/grade	Dose adjustment and management recommendations
Thrombocytopenia	
Grade 1 (≥75 × 10^9/L)	• No dose adjustment required
Grade 2 (≥50 × 10^9/L to <75 × 10^9/L)	• Dose interruption until recovery to grade ≤1. Re-initiate ribociclib at the same dose
Grade 3 (≥25 × 10^9/L to <50 × 10^9/L)	• Dose interruption until recovery to grade ≤1. Re-initiate ribociclib at the same dose level. If toxicity recurs at grade 3: temporary dose interruption until recovery to grade ≤1 and reduce ribociclib to the next lower dose level
Grade 4 (<25 × 10^9/L)	• Dose interruption until recovery to grade ≤1. Re-initiate ribociclib at the next lower dose level. If toxicity recurs at grade 4: discontinue ribociclib
ANC	
Grade 1 (≥1.5 × 10^9/L)	• No dose adjustment required
Grade 2 (≥1.0 to <1.5 × 10^9/L)	• No dose adjustment required
Grade 3 (≥0.5 to <1.0 × 10^9/L)	• Dose interruption until recovery to ≥1.0 × 10^9/L. Re-initiate ribociclib at the same dose level. If toxicity recurs at grade 3: temporary dose interruption until recovery to ≥1.0 × 10^9/L<br>• If resolved in ≤7 days, then maintain dose level<br>• If resolved in >7 days, then reduce ribociclib dose by 1 dose level<br>• For grade 3 neutropenia without fever or signs of infection on Day 14 of the first 2 cycles, consider continuing ribociclib at current dose level to complete cycle. Repeat complete blood count on Day 21. Consider dose reduction based on the neutropenia recovery (<7 days/>7 days)
Grade 4 (<0.5 × 10^9/L)	• Dose interruption until recovery to ≥1.0 × 10^9/L. Re-initiate ribociclib at the dose level below.
Febrile neutropenia	
Grade 3 (ANC <1.0 × 10^9/L with a single temperature of >38.3ºC [101ºF] or a sustained temperature of ≥38ºC [100.4ºF] for >1 hour)	• Dose interruption until improvement of ANC ≥1.0 × 10^9/L and no fever. Restart at the next lower dose level. If febrile neutropenia recurs, discontinue ribociclib
Grade 4 (life-threatening consequences, urgent intervention indicated)	• Discontinue ribociclib
Anemia (hemoglobin)	
Grade 1 (≥10.0 to LLN g/dL)	• No dose adjustment required
Grade 2 (≥8.0 to <10.0 g/dL)	• No dose adjustment required

Grade 3 (<8.0 g/dL)	• Dose interruption until recovery to grade ≤2. Re-initiate ribociclib at the same dose
Grade 4 (Life-threatening consequences, urgent intervention indicated)	• Discontinue ribociclib

ANC, absolute neutrophil count; LLN, lower limit of normal.

Online Resource 10. Ribociclib dose reduction/interruption and management recommendations for hepatic toxicities

Toxicity/grade	Dose adjustment and management recommendations
Total bilirubin without ALT/AST increase above baseline value	
Grade 1 (>ULN to 1.5 × ULN) (confirmed 48-72 hours later)	• Maintain dose level with LFTs monitored bi-weekly
Grade 2 (>1.5 to 3.0 × ULN)	• Dose interruption of ribociclib<br>• If resolved to grade ≤1 in ≤21 days, then maintain dose level<br>• If resolved to grade ≤1 in >21-28 days or toxicity recurs, then reduce by 1 dose level<br>• Repeat liver enzyme and bilirubin tests twice weekly for 2 weeks after dose resumption<br>• If toxicity reoccurs after 2 dose reductions, or recovery to grade ≤1 is >28 days, discontinue ribociclib
Grade 3 (>3.0 to 10.0 × ULN)	• Dose interruption of ribociclib, until resolved to grade ≤1, then lower by 1 dose level of ribociclib<br>• Repeat liver enzyme and bilirubin tests twice weekly for 2 weeks after dose resumption<br>• If resolved to grade ≤1 in >28 days or toxicity reoccurs, discontinue ribociclib
Grade 4 (>10.0 × ULN)	• Discontinue ribociclib
Confounding factors and/or alternative causes for increase of total bilirubin should be excluded before dose interruption/reduction. They include, but are not limited to: evidence of liver metastases; evidence of obstruction (such as elevated ALP and GGT typical of gall bladder or bile duct disease); hyperbilirubinemia due to the indirect component only (ie, direct bilirubin component ≤1 × ULN), hemolysis or Gilbert's Syndrome, other pharmacologic treatment, viral hepatitis, alcoholic or autoimmune hepatitis or other hepatotoxic drugs. For patients with Gilbert's Syndrome, dose modifications apply to changes in direct bilirubin only. Bilirubin will be fractionated if elevated.	
AST or ALT without bilirubin elevation >2 × ULN	
Same grade as baseline or increase from baseline grade 0 to grade 1 (confirmed 48-72 hours later)	• No dose adjustment required with LFTs monitored per protocol if same grade as baseline or bi-weekly in case of increase from baseline grade 0 to 1
Increase from baseline grade 0 or 1 to grade 2 (>3.0 to 5.0 × ULN)	• Dose interruption of ribociclib, if resolved to baseline grade or lower in ≤21 days, then maintain dose level<br>• If resolved to baseline grade or lower in >21 days or toxicity recurs, then reduce by 1 dose level<br>• Repeat liver enzyme and bilirubin tests twice weekly for 2 weeks after dose resumption<br>• If toxicity recurs after 2 dose reductions, or recovery to baseline grade or lower is >28 days, discontinue ribociclib

Increase from baseline grade 0 or 1 to grade 3 (>5.0 to 20.0 × ULN)	• Dose interruption of ribociclib until resolved to baseline grade or lower, then lower by 1 dose level of ribociclib<br>• Repeat liver enzyme and bilirubin tests twice weekly for 2 weeks after dose resumption<br>• If recovery to baseline grade or lower is >28 days, discontinue ribociclib<br>• If toxicity recurs, discontinue ribociclib
Increase from baseline grade 2 to grade 3 (>5.0 to 20.0 × ULN)	• Dose interruption of ribociclib until resolved to baseline grade or lower, then lower by 1 dose level of ribociclib<br>• Repeat liver enzyme and bilirubin tests twice weekly for 2 weeks after dose resumption<br>• If toxicity recurs after 2 dose reductions or recovery to baseline grade or lower is >28 days, discontinue ribociclib
Grade 4 (>20.0 × ULN)	• Discontinue ribociclib
AST or ALT and concurrent bilirubin elevation	
For patients with normal ALT and AST and total bilirubin at baseline: AST or ALT >3.0 × ULN **combined with** total bilirubin >2 × ULN without evidence of cholestasis<br>OR<br>For patients with elevated AST or ALT or total bilirubin at baseline: AST or ALT >2 × baseline AND >3.0 × ULN OR AST or ALT 8.0 × ULN (whichever is lower) **combined with** total bilirubin >2 × baseline AND >2.0 × ULN	• Discontinue ribociclib
Confounding factors and/or alternative causes for increased transaminases should be excluded before dose interruption/reduction. They include but are not limited to: concomitant medications, herbal preparations or dietary supplements, infection, hepatobiliary disorder or obstruction, new or progressive liver metastasis, and alcohol intake.	

ALT, alanine aminotransferase; ALP, alkaline phosphatase; AST, aspartate aminotransferase; GGT, gamma-glutamyl transferase; LFT, liver function test; ULN, upper limit of normal.

Online Resource 11. Ribociclib dose reduction/interruption and management recommendations for QTcF prolongation

Grade	Dose modification
All grades	• Check the quality of the ECG and the QT value and repeat if needed<br>• Perform analysis of serum electrolytes (potassium, calcium corrected for serum albumin, phosphate, magnesium). If outside of the normal range, interrupt ribociclib administration, correct with supplements or appropriate therapy as soon as possible, and repeat electrolytes until documented as normal<br>• Review concomitant medication usage for the potential to inhibit CYP3A4 and/or to prolong the QT interval<br>• Check compliance with correct dose and administration of ribociclib
Grade 1<br>(QTcF 450-480 ms)	• No dose adjustment required
Grade 2<br>(QTcF 481-500 ms)	• Interrupt ribociclib<br>• Perform a repeat ECG within 1 hour of the first QTcF of ≥481 ms.<br>• If QTcF <481 ms, restart ribociclib at the same dose. No dose adjustment required for first occurrence<br>• If QTcF remains ≥481 ms, repeat ECG as clinically indicated until the QTcF returns to <481 ms, restart ribociclib at the same dose. No dose adjustment is required for first occurrence<br>• If QTcF ≥481 ms recurs, ribociclib should be reduced by 1 dose level<br>• Repeat ECG 7 days and 14 days after dose resumption (then as clinically indicated) for any patients who had therapy interrupted due to QTcF ≥481 ms
Grade 3 (QTcF ≥501 ms on ≥2 ECGs	• Interrupt ribociclib<br>• Perform a repeat ECG within 1 hour of the first QTcF of ≥501 ms<br>• If QTcF remains ≥501 ms, consult with a cardiologist (or qualified specialist) and repeat cardiac monitoring as indicated until the QTcF returns to <481 ms<br>• If QTcF returns to <481 ms, ribociclib will be reduced by 1 dose level<br>• Repeat ECG 7 days and 14 days after dose resumption (then as clinically indicated) for any patients who had therapy interrupted due to QTcF ≥501 ms<br>• If QTcF of ≥501 ms recurs, discontinue ribociclib
Grade 4 (QT/QTcF ≥501 or >60 ms change from baseline AND torsades de pointes or polymorphic ventricular tachycardia or	• Discontinue ribociclib<br>• Seek local cardiologist (or qualified specialist) consultation and repeat cardiac monitoring as indicated until the QTcF returns to <481 ms

signs/symptoms of serious arrhythmia)	

CYP, cytochrome P450; ECG, electrocardiogram; QTcF, QT interval corrected for heart rate using Fridericia's formula.

Online Resource 12. Prohibited medications during study drug treatment

Category	Drug name
Strong CYP3A4/5 inhibitors	Boceprevir, clarithromycin, cobicistat, conivaptan, danoprevir/ritonavir, elvitegravir/ritonavir, grapefruit juice, indinavir/ritonavir, itraconazole, ketoconazole, lopinavir/ritonavir, mibefradil, nefazodone, nelfinavir, posaconazole, ritonavir, saquinavir, saquinavir/ritonavir, telaprevir, telithromycin, tipranavir/ritonavir, troleandomycin, voriconazole, indinavir, idelalisib, ombitasvir/paritaprevir, ritonavir/dasabuvir (VIEKIRA PAK).
Strong CYP3A4/5 inducers	Avasimibe,[a,b] carbamazepine, mitotane, phenobarbital, phenytoin, rifabutin, rifampin (rifampicin),[b] St. John's wort (hypericum perforatum),[b] enzalutamide.
CYP3A4/5 substrates with a narrow therapeutic index[c]	Alfentanil, apixaban (doses >2.5 mg only), aprepitant, astemizole, cisapride, cyclosporine, diergotamine, dihydroergotamine, ergotamine, fentanyl, lovastatin, nicardipine, nisoldipine, pimozide, quinidine, rivaroxaban, simvastatin, sirolimus, tacrolimus, terfenadine, thioridazine, lomitapide.
Medications with a known risk for QT prolongation[d]	Amiodarone, anagrelide, arsenic trioxide, astemizole, azithromycin, bepridil, chloroquine, chlorpromazine, cilostazol, ciprofloxacin, cisapride, citalopram, clarithromycin, disopyramide, dofetilide, domperidone, donepezil, dronedarone, droperidol, erythromycin, escitalopram, flecainide, fluconazole, halofantrine, haloperidol, ibutilide, levofloxacin, levomethadyl, mesoridazine, methadone, moxifloxacin, ondansetron (IV only), pentamidine, pimozide, probucol, procainamide, propofol, quinidine, sevoflurane, sotalol, sparfloxacin, sulpiride, terfenadine, thioridazine, vandetanib.
Herbal preparations/ medications	Herbal preparations/medications were prohibited throughout the study, including (but not limited to) St. John's wort, Kava, ephedra (ma huang), ginkgo biloba, dehydroepiandrosterone, yohimbe, saw palmetto, and ginseng.<br><br>Patients were to stop using these herbal medications 7 days prior to first dose of study drug.
Other investigational and antineoplastic therapies	Other investigational therapies could not be used while the patient was on the study.

	Anticancer therapies (ie, chemotherapy, biologic or radiation therapy, and surgery) other than the study treatments could not be given to patients while the patient was on the study medication; if such agents were required for a patient then the patient was to be discontinued from the study drug.

CYP, cytochrome P450; IV, intravenous
[a]Herbal product
[b]P-glycoprotein inducer
[c]Drugs with exposure-response indicate that increases in their exposure levels by the concomitant use of potent inhibitors may lead to serious safety concerns (eg, torsades de pointes)
[d]Source: www.crediblemeds.org (as of April 7, 2016). As far as possible, coadministration of QT-prolonging drugs or any other drugs with the potential to increase the risk of drug-related QT prolongation (eg, via a potential drug-drug interaction that increases the exposure of ribociclib or the exposure of the QT prolonging drug) was to be avoided. A definitive list of drugs with a known risk, possible risk, or conditional risk of QT prolongation and/or torsades de pointes is available online at crediblemeds.org

Online Resource 13. Treatment-related grade 5 serious adverse events (safety analysis set in core phase)

Primary system organ class	Preferred term	Number of patients
Cardiac disorders	Cardiac arrest	2 (<1)
	Acute myocardial infarction	1 (<1)
	Bradycardia	1 (<1)
	Cardiac failure congestive	1 (<1)
	Cardiac disorder	1 (<1)
	Cardiopulmonary failure	1 (<1)
	Cor pulmonale	1 (<1)
	Left ventricular dysfunction	1 (<1)
Gastrointestinal disorders	Obstruction gastric	1 (<1)
	Rectal stenosis	1 (<1)
General disorders and administration site conditions	Pyrexia	1 (<1)
	General physical health deterioration	4 (<1)
	Multiple organ dysfunction syndrome	2 (<1)
	Death	1 (<1)
	Disease progression	1 (<1)
	Organ failure	1 (<1)
	Sudden death	1 (<1)
Hepatobiliary disorders	Hepatic failure	4 (<1)
	Hepatobiliary disease	1 (<1)
Infections and infestations	Pneumonia	3 (<1)
	Sepsis	2 (<1)
	Septic shock	2 (<1)
	Staphylococcal infection	1 (<1)
	Encephalitis	1 (<1)
	Lower respiratory tract infection	1 (<1)
Injury, poisoning, and procedural complications	Toxicity to various agents	1 (<1)
Investigations	General physical condition abnormal	2 (<1)
Metabolism and nutrition disorders	Hyponatremia	1 (<1)
	Mineral metabolism disorder	1 (<1)
Neoplasms benign, malignant unspecified (including cysts and polyps)	Breast cancer	1 (<1)
	Malignant neoplasm progression	2 (<1)
	Lung neoplasm malignant	1 (<1)
	Malignant neoplasm of pleura	1 (<1)
	Breast cancer metastatic	1 (<1)
	Invasive ductal breast carcinoma	1 (<1)
Nervous system disorders	Cerebrovascular accident	2 (<1)
	Cerebral hemorrhage	1 (<1)
	Hepatic encephalopathy	1 (<1)
Renal and urinary disorders	Acute kidney injury	1 (<1)
	Renal failure	1 (<1)
Respiratory, thoracic and mediastinal disorders	Dyspnea	3 (<1)
	Pulmonary embolism	2 (<1)
	Pneumonitis	1 (<1)
	Respiratory failure	5 (<1)

	Acute respiratory failure	1 (<1)
	Interstitial lung disease	1 (<1)
	Lung infiltration	1 (<1)

Online Resource 14. Overview of adverse events of special interest in the CompLEEment-1 study

AESI, n (%)[a]	All grade	Grade 3	Grade 4	Grade 5
Neutropenia[b]	2,417 (74.5)	1,661 (51.2)	195 (6.0)	0
ALT increased	526 (16.2)	209 (6.4)	40 (1.2)	0
AST increased	459 (14.1)	159 (4.9)	25 (0.8)	0
QTcF prolongation	217 (6.7)	32 (1.0)	1 (0.0)	0

AE, adverse event; AESI, adverse event of special interest; ALT, alanine aminotransferase; AST, aspartate aminotransferase; QTcF, QT interval corrected for heart rate using Fridericia's formula.
[a]Percentage value calculated based on 3,246 patients. A patient with multiple severity grades for an AE is only counted under the maximum grade
[b]Includes "neutropenia" and "neutrophil count decreased

Online Resource 15. Mean baseline and end of treatment FACT-B scores (PRO analysis set in core phase)

FACT-B subset, mean (SD)	Baseline (n = 1,230)	End of treatment (n = 861)
Physical well-being	23.2 (4.38)	21.8 (5.18)
Social/family well-being	20.7 (5.31)	19.6 (5.78)
Emotional well-being	16.3 (4.62)	15.9 (4.98)
Functional well-being	15.9 (5.92)	15.9 (6.06)
Additional concerns	26.3 (5.55)	25.9 (6.18)
Overall	102.4 (17.78)	98.9 (20.92)

FACT-B, Functional Assessment of Cancer Therapy–Breast Cancer; PRO, patient-reported outcome; SD, standard deviation.
